# Inhibition Mechanism of *Bacillus velezensis* TS-2 Against *Streptomyces scabies*

**DOI:** 10.3390/microorganisms14102165

**Published:** 2026-09-25

**Authors:** Yudong Sun, Xuanrui Zheng, Song Liu, Lichun Wang, Weiwei Zhang, Xuepeng Fu

**Affiliations:** 1 Department of Life Science and Agroforestry, Qiqihar University, Qiqihar 161006, China; 18746085545@163.com (Y.S.); m15245802688@163.com (X.Z.); zww121@163.com (W.Z.); 2 Keshan Branch Heilongjiang Academy of Agricultural Sciences, Qiqihar 161005, China; wanglichun78@163.com; 3 State Key Laboratory of Black Soils Conservation and Utilization, Northeast Institute of Geography and Agroecology, Chinese Academy of Sciences, Harbin 150081, China; liusong@iga.ac.cn

**Keywords:** *Bacillus velezensis*, potato common scab, antibacterial mechanism, transcriptomics, *Streptomyces scabies*

## Abstract

Potato common scab is a soil-borne disease caused by *Streptomyces* spp., which affects the commodity value of potato worldwide. This study evaluated the inhibitory effect and potential mechanisms of the *Bacillus velezensis* TS-2 strain against *Streptomyces scabies*, providing a basis for developing safe and effective biocontrol agents for potato common scab. Strain TS-2 was isolated from soil adhering to the surface of healthy potato tubers. Confrontation assays, scanning electron microscopy, and pot experiments showed that strain TS-2 significantly reduced the potato common scab incidence and inhibited the mycelial growth and spore formation of *S. scabies*. Whole genome sequencing identified strain TS-2 as *Bacillus velezensis*. Transcriptome analysis showed that *B. velezensis* TS-2 significantly downregulated key genes involved in carbohydrate, lipid, and amino acid metabolism in *S. scabies*, including *aceB*, *glyA*, *acd*, thereby disrupting the core carbon and nitrogen energy supply network essential for pathogen growth and development. *B. velezensis* TS-2 also induced structural damage to the cell wall (*pbpA*), cell membrane (*ino1*) and bubble sac (*gvpA*), reducing environmental adaptability. Noticeable downregulation of genes in the *bld–whi* morphological differentiation cascade and the quorum-sensing gene *afsA* was observed under TS-2 stress, which correlates with suppressed hyphal maturation and sporulation of *S. scabies*.

## 1. Introduction

Potato common scab (PCS) is a typical soil-borne disease caused by *Streptomyces* spp. [1]. Pathogens overwinter in soil and seed potato in the form of mycelia or spores, which have both seed-borne and soil-borne characteristics, and are extremely difficult to prevent and control [2,3]. To date, multiple pathogens causing common scab have been reported worldwide including *Streptomyces scabies*, *Streptomyces turgidiscabies*, *Streptomyces acidiscabies*, and *Streptomyces bottropensis* [4,5]. Among them, *S. scabies* infects the periderm of potato tubers. Diseased tubers serve as the primary inoculum source, and the pathogen spreads in fields via soil, agricultural practices, irrigation water, and other routes [4]. As tubers expand, the lesions gradually enlarge, which markedly reduces the marketable tuber rate [4,5]. The expansion of potato cultivation has increased PCS incidence, limiting high-quality development of the potato industry.

The most direct approach to controlling PCS is to reduce the pathogen population through agricultural control, resistant varieties, chemical agents, or biological control [6,7]. However, agricultural measures cannot eliminate soil-borne pathogens, and long-term chemical use causes soil degradation and environmental pollution [8,9]. Biological control has attracted increasing attention because of its long-lasting effects, environmental friendliness, and compatibility with green agriculture [10]. Most biocontrol agents inhibit pathogens by producing secondary metabolites, showing strong application potential [11,12]. Studies have shown that *Streptomyces flavoviride* 503 reduces disease by inhibiting the synthesis of pathogenic peptidoglycan [13]. Sun et al. isolated *Bacillus velezensis* L-1 from the rhizosphere of a pear orchard, and the inhibition rate of pear ring rot was 76.55% [14]. The inhibition rate of *Bacillus velezensis* E69 isolated from rice leaves by Sha et al. to rice leaf blast was 85.97% [15]. *Bacillus velezensis* RB.DS29 isolated from the rhizosphere of black pepper by Trinh et al. could reduce the incidence of black pepper diseases [16]. Cui et al. isolated *Bacillus velezensis* 8-4 from potato tubers, which could effectively control the incidence of potato common scab and increase potato yield [17]. At present, *Bacillus velezensis* FZB42 has been commercialized and is widely used in agriculture [18].

The biocontrol mechanism of *Bacillus velezensis* mainly includes two aspects: direct bacteriostasis and indirect induced systemic resistance (ISR). The bacteria can inhibit pathogenic bacteria by producing a variety of antibiotics and siderophores [19,20,21]. *B. velezensis* CAUB946 is isolated from rice roots and produces a variety of antibiotics with broad-spectrum antibacterial effects [19]. The antifungal compounds produced by *B. velezensis* RC218 can effectively reduce the severity of the disease. Genome analysis showed that its biocontrol activity was closely related to the secretion of lipopeptides such as surfactin, fengycin and iturin [22,23]. *B. velezensis* BAC03 significantly alleviated the symptoms of tuber scab induced by *Streptomyces scabies* [24]. The strain 9912D had significant control effects on cucumber gray mold, tomato gray mold, cotton Fusarium wilt and apple rot [25]. The *B. velezensis* SQR9 mutant strain was resistant to *Pseudomonas syringae* and *Botrytis cinerea* infection [26]. *Bacillus velezensis* has a significant inhibitory effect on *Streptomyces scabies*, but its inhibitory mechanism is not clear.

Previous reports have demonstrated that some *B. velezensis* strains can suppress potato common scab caused by *S. scabies* [17,24]. However, how the phytopathogen *S. scabies* transcriptionally responds to the stress exerted by *B. velezensis* remains poorly characterized. In this study, we combined whole-genome and transcriptomic sequencing to characterize the antagonistic performance of strain TS-2, and more importantly, to dissect the global transcriptomic response of *S. scabies* under TS-2 stress. The objectives were: (i) to evaluate the antagonistic activity of TS-2 against *S. scabies*; (ii) characterize the genomic features of TS-2 via whole-genome sequencing; and (iii) reveal the genome-wide transcriptional changes of *S. scabies* upon TS-2 exposure. This work provides transcriptome-level insights for understanding interactions between *Bacillus* biocontrol agents and potato scab pathogens.

## 2. Materials and Methods

### 2.1. Cultivation of Pathogenic Bacteria

The *Streptomyces scabies* strain K37-1 used in this study was isolated from potato continuous-cropping soil in Keshan City, Heilongjiang Province, China. Species identification was conducted by multiple lines of evidence, including morphological characteristics on Gause’s synthetic medium, PCR detection of pathogenic gene txtAB, and 16S rRNA gene phylogenetic analysis (Figure S1). The strain was cultured in Gause’s synthetic medium. The specific preparation method is as follows: 20 g soluble starch (Tianjin Kaitong Chemical Reagent Co., Ltd., Tianjin, China), 1 g potassium nitrate (Liaoning Quanrui Reagent Co., Ltd., Jinzhou, China), 0.5 g dipotassium hydrogen phosphate nitrate (Tianjin Kaitong Chemical Reagent Co., Ltd., Tianjin, China), 0.5 g magnesium sulfate (Liaoning Quanrui Reagent Co., Ltd., Jinzhou, China), 0.01 g iron sulfate (Tianjin Tianli Chemical Reagent Co., Ltd., Tianjin, China) and 0.5 g sodium chloride (Tianjin Kaitong Chemical Reagent Co., Ltd., Tianjin, China) were mixed in 1 L deionized water, then an appropriate amount of agar (Beijing Aoboxing Bio-Tech Co., Ltd., Beijing, China) was added, and was autoclaved at 121 °C for 20 min. When the temperature was reduced to 60–70 °C, 30 mL of culture medium was poured into each culture dish as Gause’s synthetic medium. The *S. scabies* strain (30% glycerol cryopreservation tube) preserved in −80 °C was taken out, and 100 μL was transferred to the liquid medium of Gause’s synthetic medium under sterile conditions, and activated at 28 °C and 180 rpm for 4 d. The gradient dilution method was applied to Gause’s synthetic medium, and single colonies were obtained after inverted culture at 28 °C for 7 d. An appropriate amount of sterile water was added to the *S. scabies* culture dish to rinse the colony surface, and the eluent was collected and filtered through a three-layer sterile gauze to remove the mycelium. The concentration of spore suspension was adjusted to 1 × 10^7^ CFU·mL^−1^ and stored at 4 °C.

### 2.2. Isolation and Screening of Antagonistic Bacteria

Geocaulosphere soils were collected from healthy potato tubers in a severe potato common scab (CS) area of Keshan County, Qiqihar, China. Strains were isolated as described below. A soil sample (5 g) was suspended in 15 mL of sterile distilled water and diluted to 10^3^ to prepare the soil suspension. Finally, 100 μL of the diluted soil suspension was inoculated onto seven different microbial isolation culture media, respectively, and incubated at 28 °C for 72 h [27] (Table S1). Single colonies were picked, followed by streak plating to obtain pure single strains. A single colony was selected and inoculated into the respective liquid media and cultured at 28 °C until the OD_600_ reached 1.5 for the fermentation broth of each isolate.

The in vitro antibacterial activity of the fermentation broth of the screened strain against *S. scabies* was evaluated by confrontation test. The specific steps are as follows: *S. scabies* spore suspension (1 × 10^7^ CFU·mL^−1^) was uniformly spread on Gause’s synthetic medium (100 μL/plate). After the bacterial solution was absorbed, a sterile puncher (diameter 5 mm) was used to punch in the center of the plate, and 100 μL antagonistic strain fermentation broth and sterile LB liquid medium (control group) were added respectively. Five biological replicates were set up in each group, and the experiment was repeated three times independently. The plate was cultured in a constant temperature incubator at 28 °C for 7 d, and the bacteriostasis was observed and photographed. The diameter of the inhibition zone was measured by the cross method, and the rate of inhibition (RIR) was calculated according to the formula:

RIR(%) = TR1/CT1 × 100%

where TR1 represents the width of inhibition zone (mm), and CT1 represents the colony diameter of *S. scabies* in the control group (mm). This calculation method refers to previous studies for evaluating the antagonistic effect against spreading filamentous *Streptomyces* colonies, as conventional inhibition-zone-diameter-based formulas are poorly suited for spreading mycelial organisms.

### 2.3. Pot Experiment

Strain TS-2, which exhibited potent inhibitory activity against *Streptomyces scabies*, was screened for subsequent experimental analyses. For liquid fermentation, the strain TS-2 was inoculated into Luria-Bertani (LB) broth and incubated at 37 °C for 24 h in the dark on a rotary shaker at 180 rpm. The cell concentration of the TS-2 fermentation broth was calibrated to 1 × 10^7^ CFU·mL^−1^ and used as the working fermentation suspension for subsequent inoculation. The potato cultivar ‘Favorite 15’, a scab-susceptible variety, was adopted for the pot trials. Uniformly sized potato seed tubers were rinsed with sterile water and individually sown in pots (50 cm height × 37 cm diameter) filled with potato-cropping-free soil. The experimental soil was high-clay black soil with the following physicochemical properties: pH of 7.15 ± 0.06, electrical conductivity (EC) of 66.73 ± 1.54 μS·cm^−1^, soil moisture content of 10.82 ± 3.13%, total nitrogen of 1.29 ± 0.03 g·kg^−1^, soil organic matter of 41.93 ± 5.79 g kg^−1^, total phosphorus of 0.72 ± 0.06 g kg^−1^, total potassium of 29.14 ± 0.12 g kg^−1^, alkali-hydrolyzable nitrogen of 183 ± 11.36 mg·kg^−1^, available phosphorus of 29.71 ± 2.61 mg·kg^−1^, and available potassium of 219.30 ± 15.10 mg·kg^−1^. All potted plants were cultivated in a greenhouse under natural light with a 15 h light/9 h dark photoperiod and a temperature range of 25–30 °C. Overwatering was strictly prohibited throughout the entire cultivation period.

At the tuber formation stage, each potted potato seedling was uniformly inoculated with 50 mL of *Streptomyces scabies* spore suspension at a concentration of 1 × 10^7^ CFU·mL^−1^ into the rhizosphere soil. One week after the inoculation of *S. scabies*, 50 mL of TS-2 fermentation broth at a concentration of 1 × 10^8^ CFU·ml^−1^ was poured into the root system soils using the root drenching method. Potato seedlings inoculated with 50 mL sterilized LB culture medium were used as the control. The experimental setup consisted of three treatments: (i) *S. scabies*: pathogen *S. scabies* (control group); (ii) *S. scabies* + TS-2: *S. scabies* co-culture with strain TS-2; and (iii) LB: LB culture medium. Destructive sampling occurred after 90 d. To ensure accuracy, tubers from each potato plant were meticulously collected using an aseptic stainless-steel shovel, and any loosely attached soil was gently collected as geocaulosphere soil. Each treatment included a total of five biological replicates, with every three pots combined into one biological replicate to determine disease incidence and disease index. Disease incidence was calculated as the percentage of infected potato skins divided by the total number of potato skins in each biological replicate. Disease index was calculated based on the rating scale of 0–9 as described in [28] and calculated as follows:

Disease index = (di × li)/(L × N) × 100

where di represents the grade of disease severity and li is defined as the number of tubers at different grades of disease, L is the number of samples investigated, and N represents the highest grade nine of disease severity. The pot arrangement in the chamber was completely randomized.

### 2.4. Scanning Electron Microscopy

Strain TS-2 was subjected to a confrontation experiment with *S. scabies*. After 7 d of culture, the edge area of the *S. scabies* colony on the Petri dish (Figure S2) was selected as the sampling area, and the mycelium was scraped with a sterile surgical blade and transferred to a 1.5 mL sterile centrifuge tube. Next, 2.5% glutaraldehyde fixative was added, and fixed at 4 °C for 12 h. The fixative was discarded and rinsed with 0.1 mol·L^−1^ phosphate buffer (pH 7.0) 3 times, 15 min each time, to remove the residual fixative. The samples were dehydrated with 30%, 50%, 70%, 80%, 90%, and 95% ethanol solution for 15 min, and then dehydrated with anhydrous ethanol (100%) twice, 15 min each time, and the water was completely removed. Isoamyl acetate was added to replace ethanol, soaked for 15 min, centrifuged at 8000 rpm for 10 min, and the supernatant was discarded. Then, it was transferred to the −80 °C low temperature refrigerator for 24 h, and the ion sputtering instrument sprayed a gold coating to enhance the conductivity. The prepared samples were observed under a scanning electron microscope. The morphological differences of *S. scabies* mycelium between the control group and the treatment group were compared and analyzed, including mycelial integrity, surface roughness, spore development status, and cell wall structure.

### 2.5. Whole Genome Sequencing of Strain TS-2

A single colony of the activated TS-2 strain was picked and inoculated into optimized LB liquid medium. The strain was cultured with shaking at 38 °C and 180 rpm until reaching the mid-logarithmic growth phase (OD_600_ = 1.5). The bacterial culture was centrifuged at 8000 rpm for 10 min, and 200 mg of bacterial pellet was collected. Genomic DNA extraction and whole-genome sequencing were performed by Shanghai Meiji Biotechnology Co., Ltd. (Shanghai, China) using the Illumina NovaSeq sequencing platform. The raw sequencing data were quality-controlled using Fastp (version 0.23.4) software to remove adapter sequences, low-quality bases with a quality value lower than Q20, and short fragment reads with a length less than 50 bp, so as to obtain high-quality clean data. The optimized sequences after quality control were spliced with multiple Kmer parameters using SOAPdenovo2 (version 2.04) software to obtain the optimal contig assembly results, and local assembly optimization was further performed to construct genomic scaffolds. Genomic coding sequences were predicted using Glimmer (version 3.02), GeneMarkS (version 4.28), and Prodigal (version 2.6.3) software. The tRNAscan-SE (version 2.0) software was used for the prediction and analysis of genomic transfer RNA (tRNA), and Barrnap (version 0.9) software was applied to predict and analyze ribosomal RNA (rRNA). Corresponding databases were adopted for subsequent gene functional annotation. Based on the antiSMASH 7.1.0 online platform (https://antismash.secondarymetabolites.org/, accessed on 10 March 2025), a systematic prediction and analysis of secondary metabolite biosynthetic gene clusters in the whole genome of the TS-2 strain was carried out.

Average nucleotide identity (ANI) was calculated using FastANI (version 2.6.3) software. Digital DNA–DNA hybridization (dDDH) values were computed via the TYGS web-server (https://tygs.dsmz.de, accessed on 10 March 2025) for species-level taxonomic assignment against closely related *Bacillus* type-strain genomes.

For phylogenetic reconstruction, single-copy core housekeeping genes were extracted from TS-2 and the reference genomes. Protein sequences of housekeeping genes were aligned using MAFFT (version 7.505) software, and poorly-aligned regions were trimmed with Gblocks (version 0.91b). The concatenated alignment dataset was used for tree construction by the maximum likelihood (ML) algorithm implemented in IQ-TREE (version 2.2.0). Branch support was assessed with 1000 bootstrap replicates. Strain FJAT-46582 (GCF_002797375.1) was selected as the outgroup for rooting the phylogenetic tree.

### 2.6. B. velezensis TS-2 Extraction In Vitro Antibacterial Experiment and HPLC-MS Detection Analysis

#### 2.6.1. *B. velezensis* TS-2 Extraction In Vitro Experiment

The fermentation broth of 2 L strain TS-2 was centrifuged at 4 °C, 8000 rpm for 15 min. The supernatant was collected and vacuum filtered through 0.22 μm microporous membrane to obtain about 1.8 L of sterile fermentation supernatant, which was stored at 4 °C for later use. The crude extract was prepared by solvent extraction and acid precipitation. An equal volume of fermentation supernatant was extracted with n-butanol, ethyl acetate, and petroleum ether (1:1, *v*/*v*) in turn, and each solvent was repeatedly extracted three times. The organic phase was combined and concentrated to dryness by vacuum rotary evaporation at 45 °C to obtain the crude extract. After weighting, it was redissolved with methanol (chromatographic purity), and the reserve solution was prepared and stored at −20 °C in the dark. The fermentation supernatant was adjusted to pH 2.0 with 6 M HCl, and the protein was precipitated by standing at 4 °C for 12 h. The precipitate was collected by centrifugation at 8000 rpm and 4 °C for 15 min. The precipitate was redissolved with methanol and stored at −20 °C in the dark. In contrast, LB liquid medium without TS-2 inoculation was used as the blank control, and n-butanol, ethyl acetate, petroleum ether extract, and acid precipitate were prepared according to the same method, with methanol (chromatographic purity) as the solvent control. Each treatment was set up in 3 replicates, with methanol and the blank medium extract as the control. After 7 d of culture at 28 °C, the antibacterial effect was calculated.

#### 2.6.2. *B. velezensis* TS-2 Fractional Extract HPLC-MS Detection and Analysis

Each group of crude extract obtained by ethyl acetate and acid precipitation was placed as a 200 μL sample in a 1.5 mL centrifuge tube. Next, a 400 μL extract (methanol:acetonitrile = 1:1 (*v*:*v*)), containing 0.02 mg·mL^−1^ internal standard (L-2-chlorophenylalanine) was added; after vortex mixing for 30 s, the samples were extracted by low temperature ultrasonic extraction for 30 min (5 °C, 40 KHz) and placed at −20 °C for 30 min. After centrifugation for 15 min (13,000× *g*, 4 °C), the supernatant was removed and dried by nitrogen. Then, 100 μL of complex solution (acetonitrile:water = 1:1) was added to redissolve, vortex mixing for 30 s, and low temperature ultrasonic extraction for 5 min (5 °C, 40 KHz); after centrifugation for 10 min (13,000× *g*, 4 °C), the supernatant was transferred to the injection vial with an intubated tube for analysis. In addition, a 20 μL supernatant was taken from each sample and mixed as a quality control sample. An ultra-high performance liquid chromatography tandem Fourier transform mass spectrometry UHPLC-Exploris240 system from Thermo Fisher Scientific (Waltham, MA, USA) was used for analysis.

Chromatographic conditions: chromatographic column: ACQUITY UPLC HSS T3 (100 mm × 2.1 mm i.d., 1.8 μm; waters, Milford, CT, USA); mobile phase A was 95% water + 5% acetonitrile (containing 0.1% formic acid), and mobile phase B was 47.5% acetonitrile + 47.5% isopropanol + 5% water (containing 0.1% formic acid). The injection volume was 3 μL, and the column temperature was 40 °C.

Mass spectrometry conditions: The samples were ionized by electrospray ionization, and the mass spectrometry signals were collected by positive and negative ion scanning modes, respectively (Table S2).

### 2.7. Transcriptome Analysis of the Inhibitory Effect of B. velezensis TS-2 on S. scabies

According to the above, the confrontation experiment between strain TS-2 and *S. scabies* was carried out. After 7 d of culture, the edge area of the *S. scabies* colony on the Petri dish (Figure S2) was selected as the sampling area, and the mycelium was scraped with a sterile surgical blade and transferred to a 1.5 mL sterile centrifuge tube. After, three replicates in each group, stored at −80 °C in liquid nitrogen, were sent to the Shanghai Meiji Biomedical Technology Co., Ltd. (Shanghai, China) for sequencing analysis. Total RNA was extracted using a modified CTAB method, followed by the removal of residual genomic DNA. RNA samples were used for subsequent library construction. The RiboCop rRNA Depletion Kit for Mixed Bacterial Samples (lexogen, Greenland, NH, USA) was used to remove rRNA, mRNA was randomly broken into small fragments of about 300 bp, and double-stranded cDNA was synthesized by reverse transcription with random primers using mRNA as a template. In the synthesis of the second strand of cDNA, dUTP was used instead of dTTP for synthesis. The synthesized double-stranded cDNA was added to End Repair Mix to complement it into a flat end, 5′ end phosphorylated, and 3′ end added with an A base to connect the Y-shaped sequencing adaptor. Then, the UNG enzyme was used to eliminate the second strand of cDNA containing dUTP, so that the library contained only the first strand of cDNA. RNA library construction was performed using Illumina ^®^ Stranded mRNA Prep, Ligation of Illumina (San Diego, CA, USA). RNA-Seq double-end sequencing was performed using Illumina NovaSeq X Plus(Illumina, San Diego, CA, USA). Bioinformatics analysis was performed using data generated by the Illumina platform. All analyses were performed using the cloud platform (https://cloud.majorbio.com/) of the Shanghai Majorbio Bio-pharm Technology Co., Ltd. (Shanghai, China). During transcriptome data analysis, sequencing reads mapped to the genome of *B. velezensis* were filtered out to eliminate transcriptional interference derived from strain TS-2.

### 2.8. Screening, Annotation, Functional Enrichment Analysis, and qRT-PCR Verification of Differentially Expressed Genes

Differentially expressed genes were identified using DESeq2. Functional enrichment analysis was performed with Padj < 0.05 as the criterion for significant enrichment.

Fourteen genes were selected from the differentially expressed genes for qRT-PCR verification. The RNA polymerase beta-subunit gene rpoB, which has been widely used as a stable reference gene in gene expression studies of *S. scabies* [29], was used as the internal reference gene for data standardization, and the primers were designed using Primer-BLAST at NCBI (https://www.ncbi.nlm.nih.gov/tools/primer-blast/, accessed 17 January 2026) (Table S3). Total RNA was extracted from the *S. scabies* treatment group and control group and used for qRT-PCR (Tables S4 and S5).

### 2.9. Statistical Analysis

All in vitro antagonism assays, pot experiments, and phenotypic determinations were performed with at least three biological replicates.

Statistical calculations were conducted using SPSS 26.0 software. One-way analysis of variance (ANOVA) followed by Duncan’s multiple range test was applied to the phenotypic data. Differences were considered statistically significant at *p* < 0.05.

For transcriptome data analysis, differentially expressed genes were screened with thresholds of |log_2_FoldChange| ≥ 1 and padj < 0.05. qRT-PCR relative expression was calculated by the 2^−ΔΔCt^ method. Graphical visualization was performed using Origin 2021.

## 3. Results

### 3.1. Isolation and Screening of Antagonistic Bacteria against S. scabies

A total of 183 bacterial isolates were obtained from the geocaulosphere soils, of which three exhibited antagonistic activity against *S. scabies*. Among these three isolates, strain TS-2 exhibited the strongest antagonistic activity and was therefore selected for subsequent experiments. The antagonistic effects of the three isolates against *S. scabies* are shown in Figure S3.

### 3.2. The Inhibitory Effect of B. velezensis TS-2 on S. scabies in the Confrontation Experiment Was Studied

In order to explore the inhibitory effect of the TS-2 strain fermentation broth and cell-free fermentation filtrate on *S. scabies*, the agar column method was used for the in vitro bacteriostatic experiment. Compared with the control group (LB liquid medium), the TS-2 treatment group formed an obvious inhibition zone, with an inhibition rate of 30.53% (Figure 1). Morphological observation showed that the colony of *S. scabies* in the control group covered the Petri dishes, and the spores were grayish white. The colony edge of *S. scabies* in the treatment group was inhibited in contact with TS-2, forming an obvious transparent inhibition zone, and there was no pathogen growth in the zone.

### 3.3. The Inhibitory Effect of B. velezensis TS-2 on Potato Common Scab in the Pot Experiment

After 90 d of growth, compared with the CK-Water treatment, the CK-Disease treatment significantly increased the disease incidence (Figure 2). Compared with the CK-Disease treatment, strain TS-2 significantly reduced the disease index, but had no significant effect on disease incidence (*p* > 0.05).

### 3.4. The Effect of Strain TS-2 on the Growth of S. scabies Was Observed by Scanning Electron Microscopy

The mycelial morphology and spore development of the pathogen after co-culture were observed by scanning electron microscopy (SEM). SEM showed that the ultrastructure of *S. scabies* in the control group and the treatment group showed significant differences (Figure 3). In the control group, the mycelium morphology was complete, the surface was smooth and slightly wrinkled, septa were clearly visible, and the top of the aerial mycelium formed a typical spore chain structure. The spores were oval and the surface was rough, which is consistent with the normal development characteristics of *S. scabies*. In contrast, TS-2 treatment group showed significant morphological distortion. The mycelium at the edge of the inhibition zone was uneven in thickness, locally expanded or constricted, the transverse septum was blurred or disappeared, and no spore chain was formed at the top, indicating possible damage to the cell envelope. Although relatively smooth mycelial fragments were observed in the non-inhibitory zone, the branches were reduced and the spore production was completely inhibited, indicating that strain TS-2 had a strong inhibitory effect on the growth and sporulation of *S. scabies.* It should be noted that these observations are qualitative morphological descriptions based on micrographs without quantitative statistical analysis of the mycelial deformation or sporulation-inhibition ratios.

### 3.5. Whole Genome Sequencing Analysis of B. velezensis TS-2

#### 3.5.1. The Basic Structure and Characteristics of the Whole Genome

The genome of strain TS-2 was sequenced to further understand the mechanism of its inhibitory activity against *S. scabies* (Figure 4). The genome of TS-2 spans 3,875,104 bp, encoding 3747 protein-coding genes among a total of 4188 predicted genes, accounting for 89.46% of all genetic elements. The numbers of tRNA and rRNA were 74 and 3, respectively. There were 3747 protein-coding genes, the proportion of protein-coding genes was 89.46%, and the length of the 16S rRNA gene sequence was 1220 bp. The sequence was submitted to NCBI, and the GenBank accession number PRJNA1234876 was obtained. BLAST analysis of the 16S rRNA gene showed a >97% sequence similarity among *Bacillus velezensis*, *Bacillus amyloliquefaciens,* and *Bacillus siamensis*. Since 16S rRNA sequences cannot reliably distinguish these closely-related species, average nucleotide identity (ANI) and digital DNA–DNA hybridization (dDDH) analyses against representative reference genomes were further performed. Both the ANI and dDDH values of strain TS-2 against the type strain *Bacillus velezensis* GCF_001461825.1 satisfied the species-level assignment thresholds (ANI ≥95%, dDDH ≥70%). Phylogenetic analysis based on concatenated core housekeeping gene sequences placed TS-2 within the same clade as *B. velezensis* GCF_001461825.1 (Figure 5). Collectively, strain TS-2 was unambiguously identified as *Bacillus velezensis*.

#### 3.5.2. Prediction and Analysis of Secondary Metabolites of *B. velezensis* TS-2

Based on the antiSMASH platform (V7.1.0), the genome-wide secondary metabolite synthesis gene clusters were systematically predicted. There are 14 secondary metabolite synthesis gene clusters in the genome of the *B. velezensis* TS-2 (Table 1). Among them, except for T3PKS and the terpene-related gene clusters, the remaining nine clusters encode typical antibacterial secondary metabolites of *Bacillus*. Clusters 1, 5, 6, 9, and 13 belong to polyketide synthetic clusters; Clusters 4 and 14 are fengycin synthetic gene clusters. Cluster 6 encodes macrolactin H, Cluster 11 produces butirosin, Cluster 7 generates bacilysin, and Cluster 8 is responsible for bacillibactin biosynthesis. Cluster 10 corresponds to the surfactin synthetic gene cluster, and surfactin exhibits antibacterial and antiviral activities. Clusters 4, 8, 10, and 14 synthesize target metabolites via nonribosomal peptide synthetase pathways.

### 3.6. In Vitro Antibacterial Experiment of B. velezensis TS-2 Extraction

The in vitro antibacterial activity of different polar extracts (n-butanol, ethyl acetate, petroleum ether extract and acid precipitate) of the TS-2 fermentation supernatant against *S. scabies* was evaluated by confrontation test. After 7 d of culture, there was a significant difference in the antibacterial activity of each treatment group (*p* < 0.05) (Figure 6A), and the corresponding antagonistic phenotypes are shown in Figure 6B. The ethyl acetate extract, n-butanol extract, and acid precipitate exhibited antibacterial activity, with antibacterial rates of 38.68%, 31.07%, and 28.67%, respectively. In contrast, the antibacterial activity of the petroleum ether extract was not significantly different from that of the control group.

### 3.7. Analysis of B. velezensis TS-2 Extract by HPLC-MS

Using HPLC-MS analysis, a total of 1400 compounds were detected in the ethyl acetate extract, and 1408 compounds were detected in the acid precipitation extract. The extracts with antibacterial activity were analyzed by excluding the control group. A total of eight metabolites with antibacterial activity were annotated in ethyl acetate and acid precipitation (Table 2). The mass spectra of surfactin (C_53_H_93_N_7_O_13_), bacillaene (C_34_H_48_N_2_O_6_), and bacilysocin (C_21_H_43_O_9_P), three common antibacterial metabolites of Bacillus, were analyzed (Figure S4).

### 3.8. Transcriptome Analysis of the Inhibitory Effect of B. velezensis TS-2 on S. scabies

#### 3.8.1. Differentially Expressed Gene Analysis

Compared with the control group (CK), a total of 1720 differentially expressed genes (DEGs) were identified in the TS-2-treated group, of which 609 genes were significantly upregulated and 1111 genes were significantly downregulated (Figure 7).

#### 3.8.2. Analysis of Morphological Differentiation During the Growth Cycle of *Streptomyces scabies* Based on Downregulated Genes

**Formation of vegetative hyphae via apical growth of germinated spores**

Morphological differentiation of *S. scabies* relies on a sophisticated cytoskeleton regulatory network, in which spore germination, apical growth, and vegetative hyphal elongation require precise coordination between peptidoglycan biosynthesis and cytoskeletal proteins for cell wall construction. *Bacillus velezensis* TS-2 markedly represses the expression of pivotal genes governing morphogenesis in *S. scabies*, illuminating the molecular mechanism underlying retarded hyphal growth and development (Table 3). As a key enzyme responsible for maintaining cell wall rigidity, PbpA, together with FtsW/RodA/SpoVE family proteins functioning as peptidoglycan glycosyltransferases, peptidoglycan-binding proteins, and peptidoglycan recognition proteins, were all significantly downregulated. This observation is consistent with the massive downregulation of genes enriched in GO terms associated with cell wall, membrane, and envelope biogenesis. In addition, the actin homolog MreB and its synergistic partner DivIVA were sharply downregulated, leading to impaired synthesis of quorum-sensing signals. The concurrent reduction in *afsA* expression, together with disturbed butanoate and phenylacetate metabolism, is accompanied by the overall metabolic disorder of the pathogen under TS-2 treatment.

**Morphological Transition of Vegetative Hyphae into Aerial Hyphae**

Morphological transition from vegetative to aerial hyphae (MII transition) in *Streptomyces scabies* depends on sophisticated regulatory networks involving programmed cell death, hydrophobin biosynthesis, and quorum sensing. *Bacillus velezensis* TS-2 markedly suppressed the expression of key genes required for aerial hypha formation in *S. scabies*, revealing the molecular basis for blocked morphological differentiation cycles (Table 4). TS-2 treatment led to the decreased expression of three key hydrophobin gene clusters, which is associated with defective formation of the hydrophobic outer layer of aerial hyphae. The reduced transcription of the master developmental regulator *bldD* occurs alongside blocked aerial hyphae formation.

**Septation and Spore Chain Formation during Aerial Hyphal Development**

Aerial hyphae of *S. scabies* transform into spore chains via septation and DNA segregation, a process relying on Whi-family regulators, divisome assembly mediated by SsgA/SsgB, and the cytoskeletal protein FtsZ. TS-2 stress induces an obvious downregulation of multiple *Whi* family and sporulation-associated genes, which coincides with the complete suppression of spore chain formation observed via SEM (Table 5). Genes encoding WhiB transcriptional regulators, ssgA, ftsZ, WhiE III/IV/V/VI/VII/VIII (components of the intact WhiE polyketide synthase complex), and Dyn (a membrane-remodeling GTPase) are markedly repressed. The concurrent downregulation of these multiple components inhibits the biosynthetic machinery responsible for spore maturation markers.

#### 3.8.3. GO Annotation Analysis of Differentially Expressed Genes

In order to systematically analyze the effect of *B. velezensis* TS-2 on the biological function of *S. scabies*, Gene Ontology (GO) functional annotation analysis was performed on 1720 differentially expressed genes. The results showed that these DEGs were significantly distributed in the three GO functional categories. Among them, there were 773 biological processes (BP), accounting for 45.0%, 387 cellular components (CC), accounting for 22.5%, and 1273 molecular functions (MF), accounting for 74.0% (Figure 8A, Table S6).

#### 3.8.4. GO Enrichment Analysis of Differentially Expressed Genes

GO pathway functional enrichment analysis was performed on differentially expressed genes (DEGs). The results showed that 540 differentially expressed genes (DEGs) were enriched on 162 GO terms in the GO analysis. The top 20 GO terms with the most significant differences were the glutamine family amino acid metabolism process, arginine biosynthesis process, arginine metabolism process, α-amino acid metabolism process, organic acid metabolism process, air sac tissue, vesicle membrane, organelle membrane, carboxylic acid metabolism process, carboxylic acid metabolism process, glutamine family amino acid biosynthesis process, small molecule catabolism process, carboxylic acid catabolism process, cell modified amino acid metabolism process, small molecule metabolism process, amino acid metabolism process, amino acid betaine metabolism process, amino acid betaine biosynthesis process, and organic acid catabolism process (Figure 8B).

GO functional enrichment analysis of 202 upregulated genes was performed based on biological process (BP) and molecular function (MF). The key genes directly related to the two core mechanisms of arginine metabolic reprogramming and ABC transport system activation were screened (Table 6). TS-2 significantly induced the upregulation of key enzyme genes in the arginine biosynthesis pathway of *S. scabies*. *ArgJ*, *argB*, *argF*, *argG* and *argH* constitute a complete arginine synthase system. Significantly upregulated arginine synthase can enhance arginine synthesis and provide precursors for nitric oxide (NO) synthesis and polyamine biosynthesis. ABC transporter substrate-binding protein, ABC transporter ATP-binding protein, ABC transporter permease, and ATP-binding cassette domain-containing protein constitute a complete ABC transporter complex. The synergistic upregulation of these genes indicates that *S. scabies* attempts to expel the antibacterial substances produced by TS-2 to maintain the homeostasis of the intracellular environment.

The key genes directly related to the destruction of bubble sac structure, glutamate-nitrogen metabolism and energy metabolism were screened from 339 downregulated genes (Table 6). The downregulation of gvpA, which is the core structural protein of the vesicle, will lead to a decrease in the anaerobic adaptability of *S. scabies*. GlnA is a glutamine synthetase, and its significant downregulation will affect protein synthesis, nucleotide synthesis, and amino sugar metabolism. Simultaneous downregulation of Glu/Leu/Phe/Val dehydrogenase dimerization domain-containing protein and acyl CoA dehydrogenase, paaE involved in the phenylacetic acid metabolic pathway, and GNAT family N-acetyltransferase will cause *S. scabies* to reduce carbon source utilization and metabolic adaptability.

#### 3.8.5. KEGG Annotation Analysis of Differentially Expressed Genes

Based on the KEGG database, 1720 DEGs were functionally annotated. There were 435 differentially expressed genes significantly enriched in metabolism, accounting for 25.3%, 67 in cellular processes, 66 in environmental information processing, 54 in biological systems, and 17 in genetic information processing, which together reveal the systematic interference of TS-2 on the metabolic network of *S. scabies* (Figure 8C, Table S7). The core mechanism is reflected in the large-scale differential expression of genes related to energy metabolism, carbohydrate metabolism, and amino acid metabolism in the metabolic pathway, the significant enrichment of terpenoids, polyketide metabolism, and exogenous compound biodegradation pathways, and the differential expression of genes related to membrane transport and signal transduction pathways, indicating that the basic energy supply and material synthesis ability of *S. scabies* are seriously impaired.

#### 3.8.6. KEGG Enrichment Analysis of Differentially Expressed Genes

KEGG enrichment analysis showed that 713 DEGs were significantly enriched in 172 metabolic pathways, of which the top 20 most significant pathways were focused on amino acid metabolism, sulfur and nitrogen cycle disorders, antibiotic synthesis and resistance response, and energy metabolism disorders. These four functional modules systematically reveal the molecular inhibition mechanism of TS-2 on *S. scabies* (Figure 8D). The blocked amino acid metabolism was reflected in the significant enrichment of glycine–serine–threonine metabolism, alanine–aspartate–glutamic acid metabolism, and the arginine biosynthesis and lysine degradation pathways, which was confirmed by the blocked amino acid metabolism of the glutamate family and the imbalance of arginine synthesis and decomposition in the GO analysis, indicating disorder of the nitrogen metabolism hub and a supply crisis of protein synthesis precursors.

KEGG pathway enrichment analysis was performed on 104 upregulated DEGs, and key genes directly related to the four core mechanisms of sulfur metabolism activation, ABC transport system resistance response, arginine biosynthesis, and non-ribosomal peptide synthesis were screened. PLP is a dependent cysteine synthetase, and the sulfate adenylyltransferase subunit is an inorganic sulfur reduction assimilation pathway (Table 7). Synergistic upregulation of the above genes indicated that *S. scabies* enhanced sulfate reduction and cysteine synthesis, providing precursors for glutathione biosynthesis. Synchronously upregulated thiC was involved in the thiamine (vitamin B1) pyrophosphate (TPP) synthesis pathway, DrrA family proteins, argG and argC, indicating that *S. scabies* enhanced arginine de novo synthesis and provided substrates for nitric oxide (NO) synthesis and polyamine accumulation, thereby maintaining oxidation reactions and nucleic acid stability.

KEGG pathway enrichment analysis was performed on 253 downregulated DEGs, and key genes directly related to the four core mechanisms of one-carbon unit metabolism, TCA-glyoxylic acid cycle blocking, inositol signal metabolism inhibition and folic acid synthesis blocking were screened (Table 7). GlyA hydroxymethyltransferase, aceB glyoxylate cycle key enzyme, ino1, folP, caspase family protein, caspase family protein and the paaE phenylacetic acid degradation pathway were significantly downregulated.

### 3.9. qRT-PCR Validation of Differentially Expressed Genes

qRT-PCR was performed to detect the expression levels of differential genes and verify the reliability of transcriptome data, with rpoB as the internal reference gene and three biological replicates for each target gene. The expression trends of 11 out of the 14 detected genes were highly consistent with the transcriptome results (Table 8).

## 4. Discussion

In this study, the in vitro antagonism assay, pot trial, scanning electron microscopy, and multi-omics approaches were integrated to systematically clarify the inhibitory mechanism of *Bacillus velezensis* TS-2 against *Streptomyces scabies*. The fermentation broth of TS-2 exhibited an inhibition rate of 30.53% in the plate confrontation test, and pot experiments verified that TS-2 could significantly reduce the disease index of potato common scab, which is consistent with previous reports on the antagonistic activity of *B. velezensis* against scab-causing streptomycetes [24]. SEM observation revealed obvious mycelial deformation, blurred septa, and the fully blocked spore chain formation of *S. scabies* under TS-2 treatment, which is consistent with the inhibitory phenotypes reported for lipopeptide metabolites of *Bacillus* strains [22,30].

Whole-genome sequencing identified strain TS-2 as *B. velezensis*, whose genome size and GC content were highly consistent with model strains FZB42 and SQR9 [31,32]. A total of 14 secondary metabolite biosynthetic gene clusters were predicted via the antiSMASH platform. Surfactin, bacillaene, and bacilysocin were detected by combined solvent extraction and HPLC-MS analysis. These compounds represent potential antibacterial metabolites encoded by the corresponding biosynthetic gene clusters of TS-2, which is consistent with previous metabolomic reports on *B. velezensis* [33].

Transcriptomic data illustrated that TS-2 drastically downregulated key metabolic genes including *aceB*, *glyA,* and *glnA*, disturbing the carbon and nitrogen central metabolic network and hindering the material and energy supply required for the normal growth and morphological differentiation of pathogens. To cope with toxicity stress, *S. scabies* upregulated genes involved in arginine synthesis and ABC transporters for compensation, which led to continuous intracellular energy exhaustion, and similar metabolic disorders have been documented in studies of *B. velezensis* against soil-borne pathogens [34,35]. TS-2 also suppressed the expression of structural genes *pbpA*, *ino1*, and *gvpA*, destroying the integrity of the cell wall, cell membrane, and gas vesicles, and weakening the environmental adaptability of pathogens. Genes belonging to the *bld*, *Whi,* and *SsgA* developmental regulatory families exhibited reduced transcription under TS-2 stress, coinciding with retarded mycelial growth and suppressed sporulation. The transcriptomic results were consistent with the SEM phenotypes, and qRT-PCR further validated the reliability of RNA-Seq data for most differentially expressed genes.

Most previous relevant studies only focused on single antibacterial compound or individual regulatory pathway, while this study integrated genomic, metabolomic, and transcriptomic data to elaborate the multi-target synergistic inhibition mechanism of TS-2. Limitations still exist in this work: only indoor and pot assays were carried out without multi-site field verification, and single purified metabolites were not tested to clarify their independent antibacterial efficacy. In addition, this study only analyzed the overall transcriptional and metabolic response of *S. scabies* under the stress of *Bacillus velezensis* TS-2 at the transcriptome level, and no targeted detection of thaxtomin toxin and its biosynthetic genes was performed. Targeted verification experiments will be carried out in follow-up research to further explore the inhibitory effect of TS-2 on pathogen toxin synthesis. Further field efficacy trials and target verification of isolated active compounds should be conducted in subsequent research [35,36], so as to provide comprehensive theoretical support for biocontrol agent development. In conclusion, *B. velezensis* TS-2 possesses moderate biocontrol potential and can serve as a promising strain resource for the green management of potato common scab.

## 5. Conclusions

This study confirmed that *Bacillus velezensis TS-2* possesses moderate biocontrol potential against potato common scab. The confrontation experiment showed that the inhibition rate of the fermentation broth against the pathogen was 30.53%. The pot experiment also confirmed that strain TS-2 could significantly reduce the disease index of potato common scab. Scanning electron microscope observations showed that strain TS-2 could cause morphological distortion and structural damage of *Streptomyces scabies* mycelium and completely inhibit its spore production. The whole genome sequencing confirmed that the strain TS-2 was *Bacillus velezensis*, and the genetic characteristics were consistent with the characteristics of Bacillus. HPLC-MS analysis detected surfactin, bacillaene, and bacilysocin in the TS-2 fermentation extracts; these compounds are potential antibacterial metabolites predicted by the secondary metabolite biosynthetic gene clusters of strain TS-2. Transcriptome analysis showed that TS-2 repressed core carbon and nitrogen metabolic genes, impaired the cell wall, membrane, and gas vesicle structures, and reduced the transcription of morphological differentiation and quorum-sensing related genes, jointly weakening the environmental adaptability of *S. scabies*. Collectively, *B. velezensis* TS-2 exhibits moderate antagonistic activity against *S. scabies* by interfering with pathogen central metabolism, cell structural integrity, and morphological differentiation processes, which provides a theoretical reference for developing biocontrol resources to control potato common scab.

## Figures and Tables

**Figure 1:**
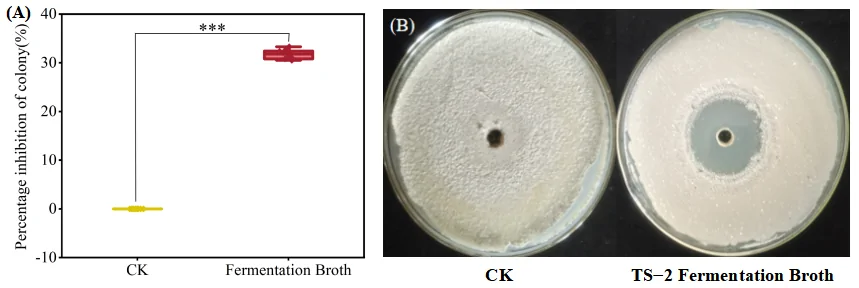
The inhibitory effect of strain TS-2 fermentation broth on *S. scabies* in vitro. (**A**) Statistical analysis of inhibition rate. (**B**) Images of antagonistic effect. CK: blank control; TS-2 Fermentation Broth: TS-2 treatment. Different letters indicate significant differences (*** *p* < 0.001).

**Figure 2:**
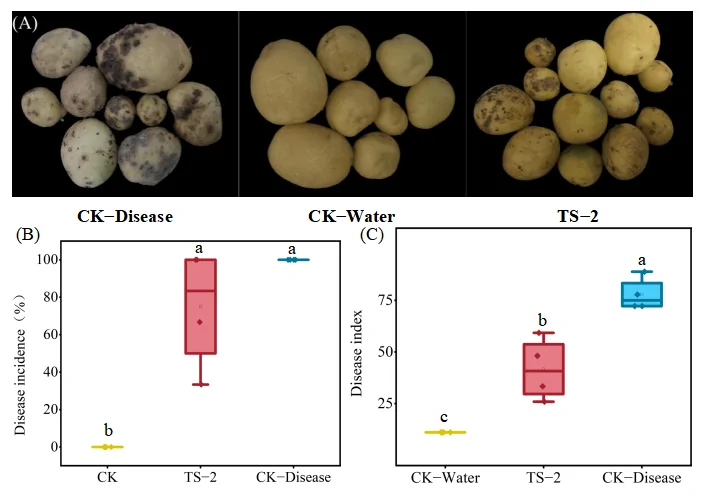
The results of pot experiment. (**A**) Symptoms of potato tubers in different treatments. (**B**) Disease incidence. (**C**) Disease index. CK-Water: only inoculated with sterile water; CK-Disease: only inoculated with the pathogen spore suspension; TS-2: *Bacillus velezensis* TS-2 and the pathogen spore suspension were inoculated. Different letters indicate significant difference (*p* < 0.05).

**Figure 3:**
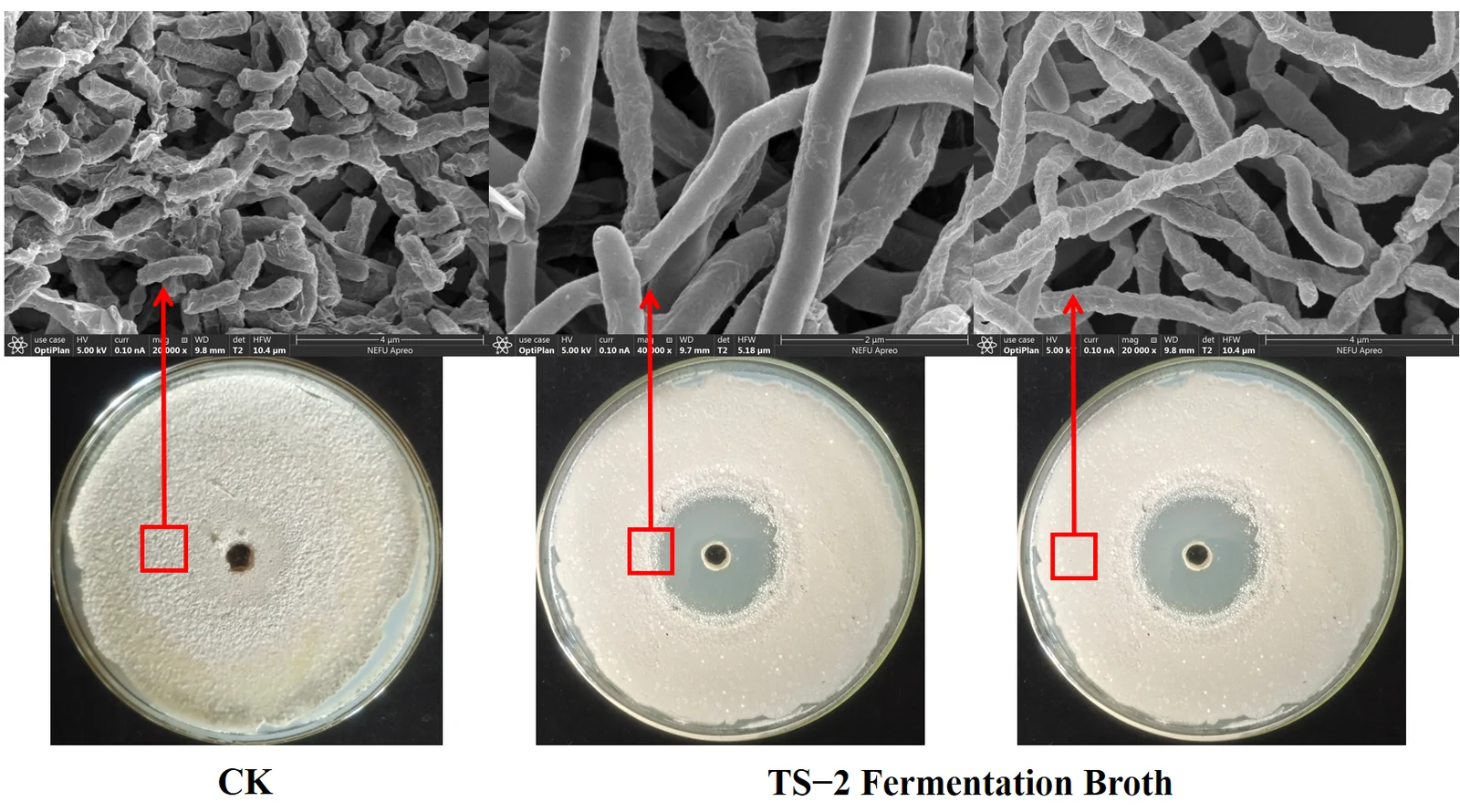
Effects of TS-2 on the hyphal morphology of *S. scabies* observed by SEM. CK: Control with intact hyphae, clear septa, and normal spore chains; TS-2: Deformed, damaged hyphae with completely inhibited sporulation. Red boxes and arrows indicate the observation areas of the mycelial morphology (SEM) and the colony morphology of the CK and TS-2 fermentation broth groups.

**Figure 4:**
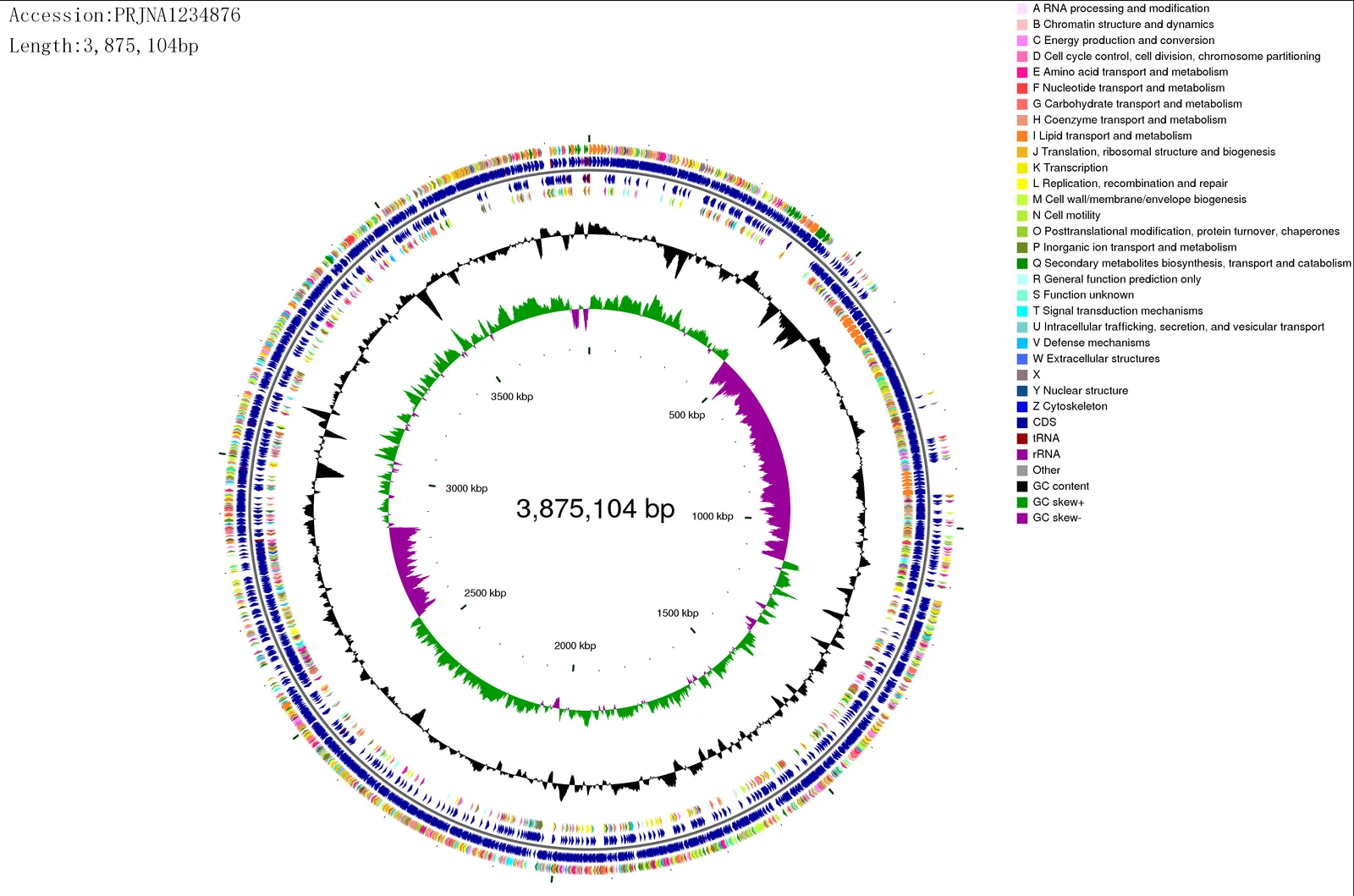
Circular genome map of Bacillus velezensis TS-2 showing the genome structure, CDS, tRNA, rRNA, GC content, and GC skew.

**Figure 5:**
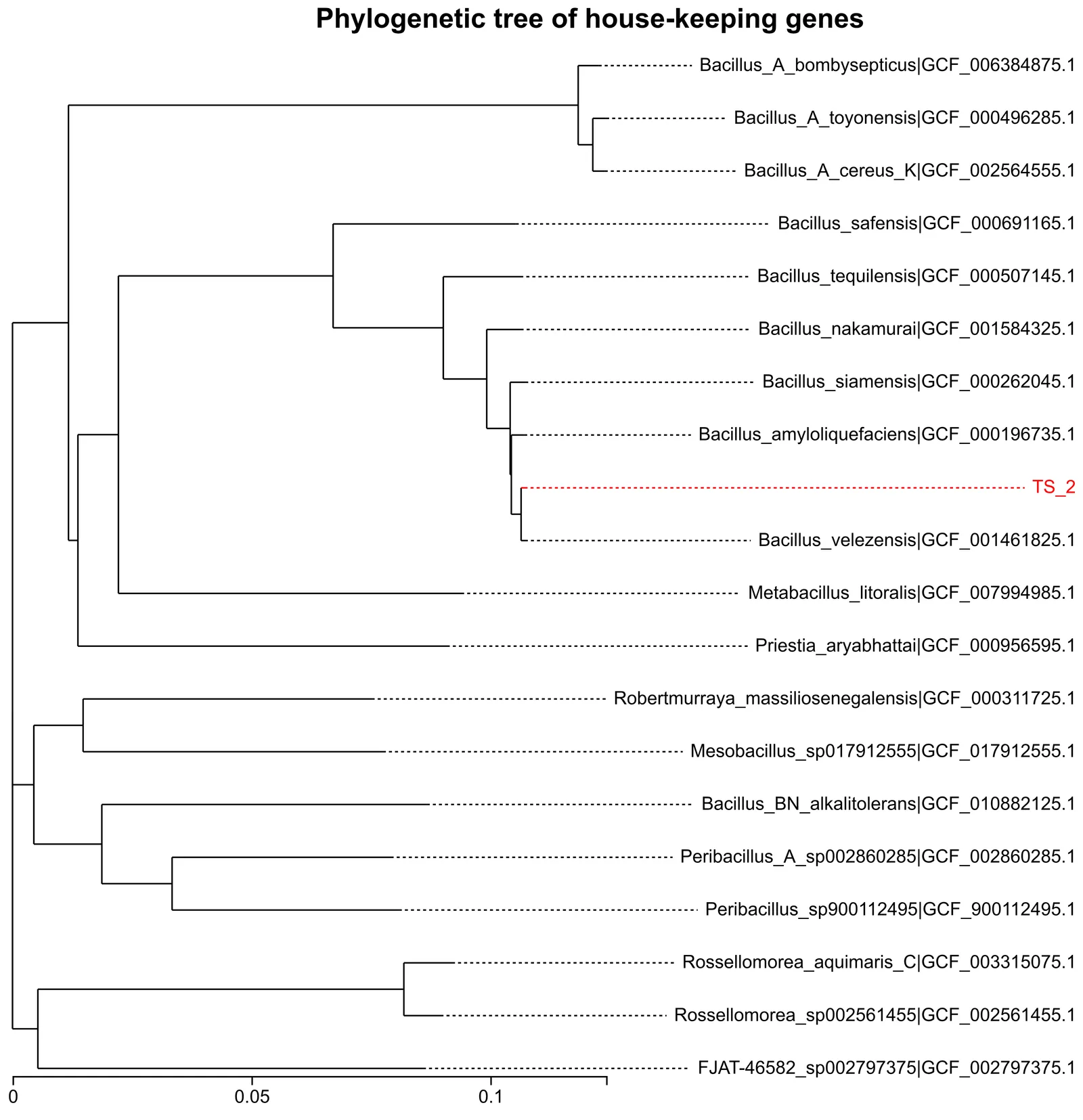
Phylogenetic tree based on concatenated core housekeeping gene sequences showing the phylogenetic position of strain TS-2 among related *Bacillus* species. The maximum-likelihood phylogenetic tree was constructed using IQ-TREE based on concatenated core housekeeping gene sequences. Bootstrap analysis was performed with 1000 replicates. Strain TS-2 is highlighted in red. GenBank assembly accession IDs of reference genomes are labeled after each strain. Strain FJAT-46582 (GCF_002797375.1) was selected as the outgroup for tree rooting. The scale bar represents the number of substitutions per site.

**Figure 6:**
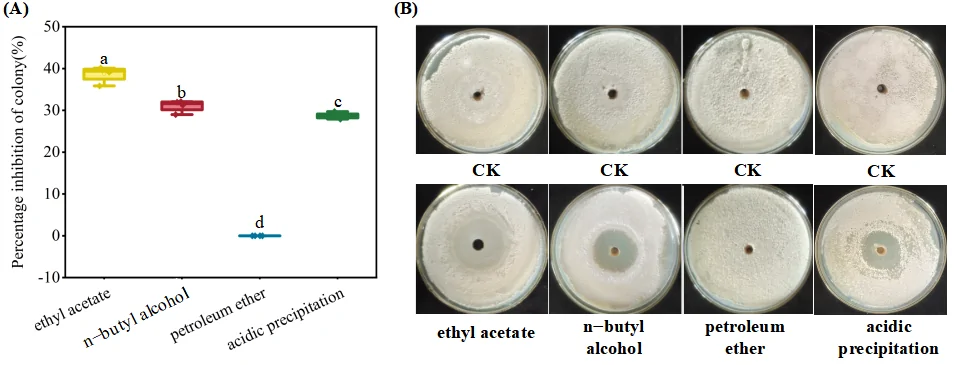
Antibacterial activity of different solvent extracts from the TS-2 fermentation supernatant. (**A**) Statistical analysis of inhibition rates. (**B**) Images of in vitro antibacterial effect. Different letters indicate significant differences (*p* < 0.05).

**Figure 7:**
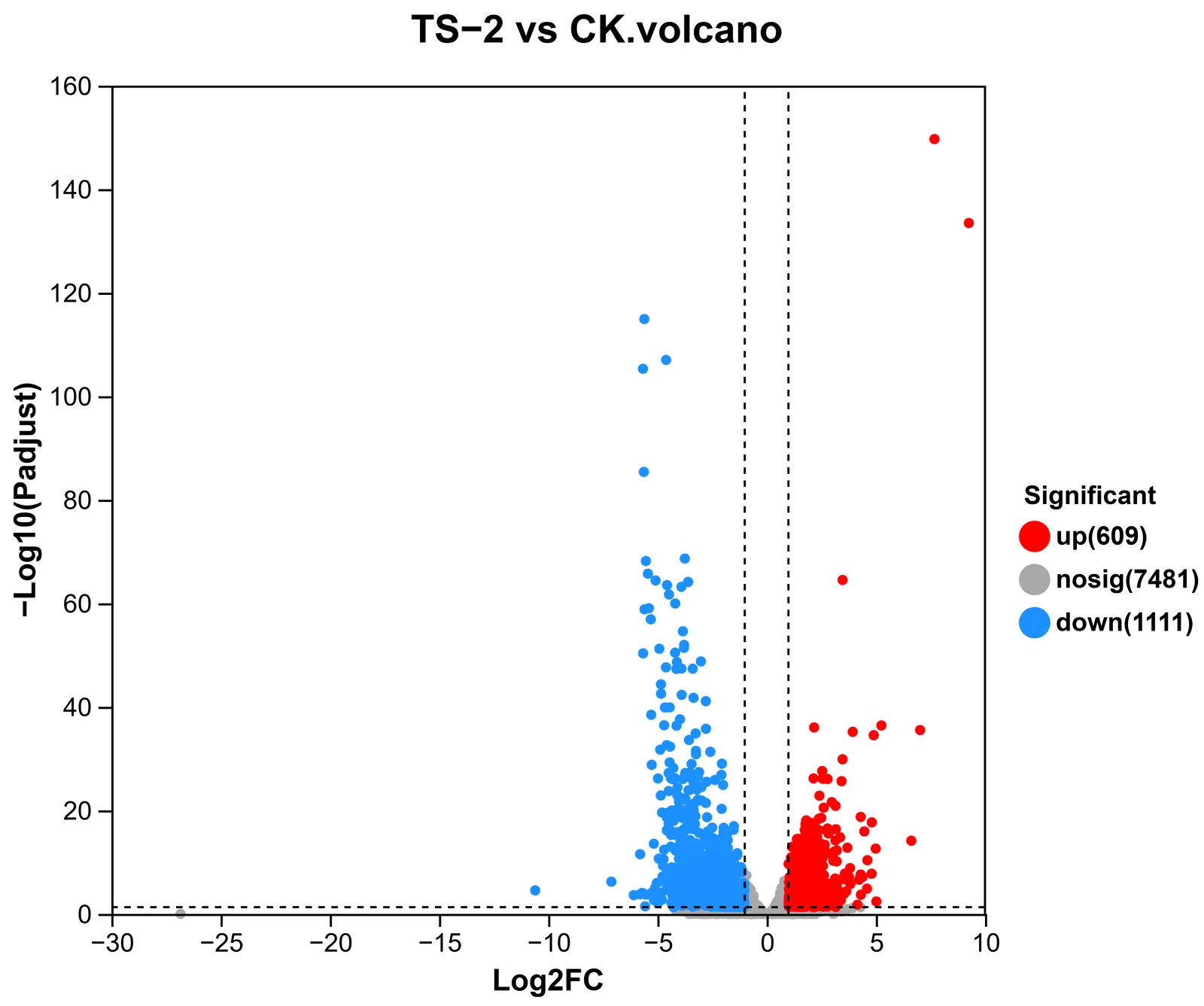
Volcano plot of differentially expressed genes (DEGs) in *Streptomyces scabies* between the control and TS-2 treatment groups. The abscissa is the fold of the difference in the expression of the gene between the two samples. The ordinate is the statistical test value of the difference in gene expression, that is, the *p* value. Red dots represent genes significantly upregulated, blue dots indicate genes significantly downregulated, and gray dots indicate non-significantly differential genes.

**Figure 8:**
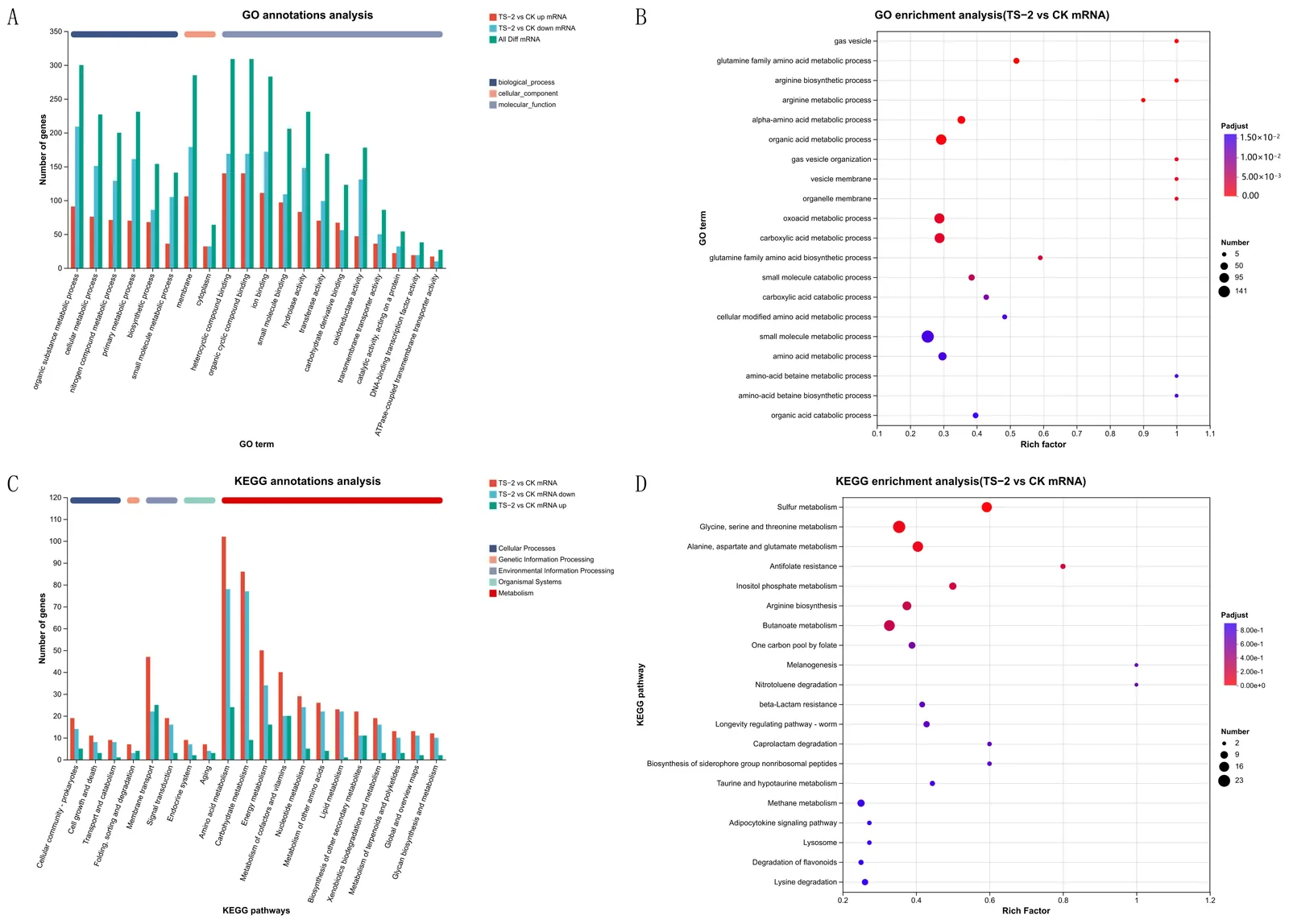
(**A**) GO annotation of differentially expressed genes. Distribution of biological process, cellular component, and molecular function. (**B**) GO enrichment analysis of differentially expressed genes. Top 20 significantly enriched GO terms are displayed. (**C**) KEGG annotation of differentially expressed genes. Main metabolic and cellular process categories are shown. (**D**) KEGG enrichment analysis of differentially expressed genes. The top 20 significantly enriched metabolic pathways are displayed.

**Table 1:** Prediction of TS-2 strain secondary metabolites.

Cluster	Type	Location	Secondary Metabolites	Function	GeneclusterID Similarity
cluster 1	transAT-PKS-like	1-45835	Difficidin	Antibacterial	BGC0000176 (53%)
cluster 2	T3PKS	207046-246472	-	-	-
cluster 3	Terpene	297255-319139	-	-	-
cluster 4	NRPS	340755-465356	Fengycin	Antifungal	BGC0001095 (100%)
cluster 5	transAT-PKS	554490-655063	Bacillaene	Antifungal, antibacterial	BGC0001089 (100%)
cluster 6	transAT-PKS	874228-962444	Macrolactin H	Antifungal, antibacterial, antivirus	BGC0000181 (100%)
cluster 7	Other	291386-332805	Bacilysin	Antibacterial	BGC0001184 (100%)
cluster 8	RiPP-like	852704-904493	Bacillibactin	Accumulates and takes up iron ions antivirus,	BGC0000309 (100%)
cluster 9	transAT-PKS	428614-462740	Difficidin	Antibacterial	BGC0000176 (46%)
cluster 10	NRPS	115116-142942	Surfactin	Antibacterial, antivirus	BGC0000433 (43%)
cluster 11	PKS-like	184800-226045	Butirosin A/Butirosin B	Antibacterial	BGC0000693 (7%)
cluster 12	RRE-containing	10796-33974	Plantazolicin		BGC0000569 (91%)
cluster 13	transAT-PKS-like	1-23521	Difficidin	Antibacterial	BGC0000176 (26%)
cluster 14	NRPS	1-9395	Fengycin	Antifungal	BGC0001095 (13%)

Note: “-”: indicates unknown.

**Table 2:** Antimicrobial metabolites.

Metabolite	CAS	M/Z
Cerulenin	17397-89-6	222.1134417
Surfactin	24730-31-2	1036.688566
Surfactin B	-	1022.673046
Bacillaene	-	581.3574275
Bacilysocin	-	469.256734
Itaconic Acid	97-65-4	129.0192305
Surfactin A	-	1028.62623
Surfactin C2	-	1034.674986

**Table 3:** Differentially expressed genes related to cell-wall biosynthesis and cytoskeleton regulation under TS-2 stress.

Gene Name	Gene Description	Log_2_^FC^(TS-2/CK)	*p* Value	Regulate
SCAB_RS14030	FtsW/RodA/SpoVE	−2.90970267827	9.30654096034 × 10^−18^	Down
SCAB_RS30305	Rod shape-determining protein (MreB)	−4.18837964588	1.0570492077 × 10^−53^	Down
SCAB_RS28665	Rod shape-determining protein (MreB)	−1.17873108536	2.46648561936 × 10^−6^	Down
SCAB_RS27375	Peptidoglycan-binding protein	−3.343599745	8.08809511743 × 10^−45^	Down
SCAB_RS31990	Peptidoglycan recognition protein	−3.304718909	6.13221535296 × 10^−24^	Down
SCAB_RS03880	Peptidoglycan-binding protein	−1.225583476	6.1191324351 × 10^−5^	Down
SCAB_RS14035	Penicillin-binding transpeptidase domain-containing protein (pbpA)	−2.378541669	8.00191796828 × 10^−12^	Down
SCAB_RS05740	ScbA/BarX family gamma-butyrolactone biosynthesis protein(AfsA)	−1.330486471	2.01722990585 × 10^−7^	Down

**Table 4:** Differentially expressed genes associated with aerial-hyphae formation and morphological differentiation of Streptomyces scabies.

Gene Name	Gene Description	Log_2_^FC^(TS-2/CK)	*p* Value	Regulate
SCAB_RS45825	SapB/AmfS family	−2.013547421	1.55640089284 × 10^−6^	Down
chpG	Chaplin ChpG	−3.656812557	1.91579155726 × 10^−20^	Down
SCAB_RS28165	Chaplin	−3.823775269	1.48095374329 × 10^−16^	Down
SCAB_RS28170	Chaplin	−4.481733846	6.10875311796 × 10^−20^	Down
SCAB_RS28275	Chaplin	−2.206311116	9.72092351521 × 10^−13^	Down
SCAB_RS28150	Rodlin	−4.691880153	1.20437801489 × 10^−14^	Down
SCAB_RS28160	Rodlin	−4.347386108	4.51439458867 × 10^−11^	Down
SCAB_RS28155	Rodlin	−4.573095035	1.24792880133 × 10^−10^	Down
bldD	BldD	−1.063810792	1.51360993323 × 10^−5^	Down
SCAB_RS05740	ScbA/BarX/family(AfsA)	−1.330486471	2.01722990585 × 10^−7^	Down
chpG	Chaplin ChpG	−3.656812557	1.91579155726 × 10^−20^	Down

**Table 5:** Differentially expressed genes involved in spore-chain formation and sporulation regulation under TS-2 stress.

Gene Name	Gene Description	Log_2_^FC^(TS-2/CK)	*p* Value	Regulate
SCAB_RS20615	whiEVI	−5.524835241	6.8876237097 × 10^−72^	Down
SCAB_RS20600	whiEIII	−5.431930503	2.18629725223 × 10^−69^	Down
SCAB_RS20595	whiEII	−5.08086523	5.47440546441 × 10^−68^	Down
SCAB_RS20605	whiEIV	−5.587138021	3.36610385699 × 10^−62^	Down
SCAB_RS20620	whiEVII	−5.652960732	1.48526594968 × 10^−53^	Down
SCAB_RS20585	whiEVIII	−4.33477075	1.45665591869 × 10^−22^	Down
SCAB_RS20610	whiEV	−5.161106919	7.41123507781 × 10^−16^	Down
SCAB_RS17165	WhiB family transcriptional	−2.579733403	1.08302317044 × 10^−14^	Down
SCAB_RS05910	WhiB family transcriptional	−2.837786487	0.000123087900456	Down
SCAB_RS28285	dynamin family protein	−2.732988038	1.44867174976 × 10^−15^	Down
SCAB_RS35735	SsgA family sporulation	−3.383539546	9.58411906321 × 10^−22^	Down
SCAB_RS25450	SsgA family sporulation	−1.612324887	1.05655822303 × 10^−5^	Down
SCAB_RS26905	SsgA family sporulation	−3.260497976	6.42884563214 × 10^−14^	Down

**Table 6:** GO functional enrichment analysis of DEGs.

Gene ID	Gene Name	Gene Description	Regulate
SCAB_RS35520	argJ	Bifunctional glutamate N-acetyltransferase/amino-acid acetyltransferase ArgJ	Up
SCAB_RS35525	argB	Acetylglutamate kinase	Up
SCAB_RS20980	argG	Argininosuccinate synthase	Up
SCAB_RS35565	argH	Argininosuccinate lyase	Up
SCAB_RS10940	argF	Ornithine carbamoyltransferase	Up
SCAB_RS05935	SCAB_RS05935	ABC transporter substrate-binding protein	Up
SCAB_RS27575	SCAB_RS27575	ABC transporter substrate-binding protein	Up
SCAB_RS19060	SCAB_RS19060	ABC transporter ATP-binding protein	Up
SCAB_RS29870	SCAB_RS29870	ABC transporter ATP-binding protein	Up
SCAB_RS31695	SCAB_RS31695	ATP-binding cassette domain-containing protein	Up
SCAB_RS21515	SCAB_RS21515	Daunorubicin resistance protein DrrA family ABC transporter ATP-binding protein	Up
SCAB_RS21520	SCAB_RS21520	ABC transporter permease	Up
SCAB_RS25895	SCAB_RS25895	ABC transporter permease	Up
SCAB_RS49055	SCAB_RS49055	ABC transporter permease	Up
SCAB_RS08160	SCAB_RS08160	Class I SAM-dependent methyltransferase	Up
SCAB_RS08210	SCAB_RS08210	Gas vesicle structural protein GvpA	Down
SCAB_RS18245	SCAB_RS18245	Gas vesicle structural protein GvpA	Down
SCAB_RS35350	SCAB_RS35350	Glutamine synthetase family protein	Down
SCAB_RS06065	SCAB_RS06065	Glu/Leu/Phe/Val dehydrogenase dimerization domain-containing protein	Down
SCAB_RS27745	SCAB_RS27745	Thiamine pyrophosphate-binding protein	Down
SCAB_RS06065	SCAB_RS06065	Glu/Leu/Phe/Val dehydrogenase dimerization domain-containing protein	Down
SCAB_RS35350	SCAB_RS35350	Glutamine synthetase family protein	Down
SCAB_RS39370	paaE	1%2C2-phenylacetyl-CoA epoxidase subunit PaaE	Down
SCAB_RS40150	SCAB_RS40150	Acyl-CoA dehydrogenase family protein	Down
SCAB_RS04975	SCAB_RS04975	GNAT family N-acetyltransferase	Down
SCAB_RS40435	SCAB_RS40435	Acyl-CoA dehydrogenase family protein	Down

**Table 7:** KEGG Functional Enrichment Analysis of DEGs.

Gene ID	Gene Name	Gene Description	Regulate
SCAB_RS42930	SCAB_RS42930	PLP-dependent cysteine synthase family protein	Up
SCAB_RS09375	SCAB_RS09375	Sulfate adenylyltransferase subunit 1	Up
SCAB_RS31695	SCAB_RS31695	ATP-binding cassette domain-containing protein	Up
SCAB_RS21515	SCAB_RS21515	Daunorubicin resistance protein DrrA family ABC transporter ATP-binding protein	Up
SCAB_RS22010	thiC	Phosphomethylpyrimidine synthase ThiC	Up
SCAB_RS20980	argG	Argininosuccinate synthase	Up
SCAB_RS35515	argC	N-acetyl-gamma-glutamyl-phosphate reductase	Up
SCAB_RS00670	SCAB_RS00670	Non-ribosomal peptide synthetase	Up
SCAB_RS00665	SCAB_RS00665	Non-ribosomal peptide synthetase	Up
SCAB_RS13685	glyA	Serine hydroxymethyltransferase	Down
SCAB_RS03190	glyA	Serine hydroxymethyltransferase	Down
SCAB_RS13035	glyA	Serine hydroxymethyltransferase	Down
SCAB_RS38540	SCAB_RS38540	Caspase family protein	Down
SCAB_RS08680	aceB	Malate synthase A	Down
SCAB_RS21865	SCAB_RS21865	Inositol-3-phosphate synthase	Down
SCAB_RS39370	paaE	1%2C2-phenylacetyl-CoA epoxidase subunit PaaE	Down
SCAB_RS03220	folP	Dihydropteroate synthase	Down

**Table 8:** qRT-PCR validation of key genes in *S. scabies* under TS-2 treatment, with rpoB as the internal reference gene.

GeneID	Genes Description	Expression	log2 Fold-Change qRT-PCR	log2 Fold-Change RNA-Seq
SCAB_RS03355	RNA polymerase sigma factor	-	−0.993 ± 0.229	+4.81819661007
SCAB_RS19060	ABC transporter ATP-binding protein	-	−3.899 ± 0.916	+3.43357269047
SCAB_RS20980	Argininosuccinate synthase	Up	+2.150 ± 0.005	+1.77847763932
SCAB_RS29870	ABC transporter ATP-binding protein	Up	+1.68 ± 0.262	+1.79221034049
SCAB_RS36015	Transcriptional regulator BldD	Down	−0.782 ± 0.143	−1.06381079246
SCAB_RS41120	SigB/SigF/SigG family RNA polymerase sigma factor	Down	−2.730 ± 0.151	−2.71876132996
SCAB_RS17165	WhiB family transcriptional regulator	Down	−2.468 ± 0.102	−2.57973340255
SCAB_RS14030	FtsW/RodA/SpoVE family cell cycle protein	Down	−2.178 ± 0.502	−2.90970267827
SCAB_RS20620	TcmI family type II polyketide cyclase	Down	−5.332 ± 0.079	−5.65296073241
SCAB_RS38735	LuxR C-terminal-related transcriptional regulator	Down	−2.121 ± 0.103	−3.29149189605
SCAB_RS20595	Cupin domain-containing protein	Down	−0.073 ± 0.166	−5.08086522964
SCAB_RS37175	Elongation factor Tu	-	+4.946 ± 0.029	−5.25655757078
SCAB_RS05740	ScbA/BarX family gamma-butyrolactone biosynthesis	Down	−0.710 ± 0.049	−1.33048647059
SCAB_RS35735	SsgA family sporulation/cell division	Down	−3.369 ± 0.165	−3.38353954645

Note: Up and Down represent identical expression trends between qRT-PCR and RNA-Seq; “-” represents inconsistent trends.

## Data Availability

The original data presented in the study are openly available in the NCBI Sequence Read Archive (SRA) under the BioProject accession number PRJNA1495439 (https://www.ncbi.nlm.nih.gov/bioproject/PRJNA1495439, accessed on 15 July 2026). The whole-genome sequence assembly of strain TS-2 has been deposited in NCBI GenBank under the BioProject accession number PRJNA1234876 (https://www.ncbi.nlm.nih.gov/bioproject/PRJNA1234876, accessed on 12 March 2025). Other relevant data can be provided upon request.

## Supplementary Materials

The following supporting information can be downloaded at: https://www.mdpi.com/article/10.3390/microorganisms14102165/s1, Figure S1. Identification of the pathogenic strain *S. scabies* K37-1. (A) PCR amplification of the pathogenicity gene txtAB; (B) 16S rRNA gene-based phylogenetic tree. Supplementary Table S1. Medium formulation. Supplementary Figure S2: The schematic diagram of scanning electron microscope and transcriptomics sampling site. The red part is the sampling area. Supplementary Table S2. Mass Spectrometric Parameters. Supplementary Table S3. Genes and primers. Supplementary Table S4. qRT-PCR System. Supplementary Table S5. qRT-PCR amplification conditions. Supplementary Figure S3. Antagonistic activities of the antagonistic isolates TS-1, TS-2, and TS-3 against S. scabies in confrontation assays. CK: control without antagonistic bacterium. Supplementary Figure S4. Mass Spectra of Surfactin, Bacillaene, and Bacilysocin. (A–F) Represent the mass spectra of Surfactin, Surfactin A, Surfactin B, Surfactin C2, Bacillaene, and Bacilysocin, respectively. Supplementary Table S6. GO Annotation of DEGs. Supplementary Table S7. KEGG Annotation of DEGs.
