# Supplementary material for: Inhibition Mechanism of Bacillus velezensis TS-2 Against Streptomyces scabies
Source: Microorganisms. 2026 Sep 25;14(10):2165. doi: 10.3390/microorganisms14102165 (PMC13707432; doi:10.3390/microorganisms14102165)
Supplement: Supplementary file 1 [file microorganisms-14-02165-s001.zip › microorganisms-4490995-supplementary.pdf]

Supplementary Material

1 Supplementary Figures and Tables

1.1 Supplementary Figure S1

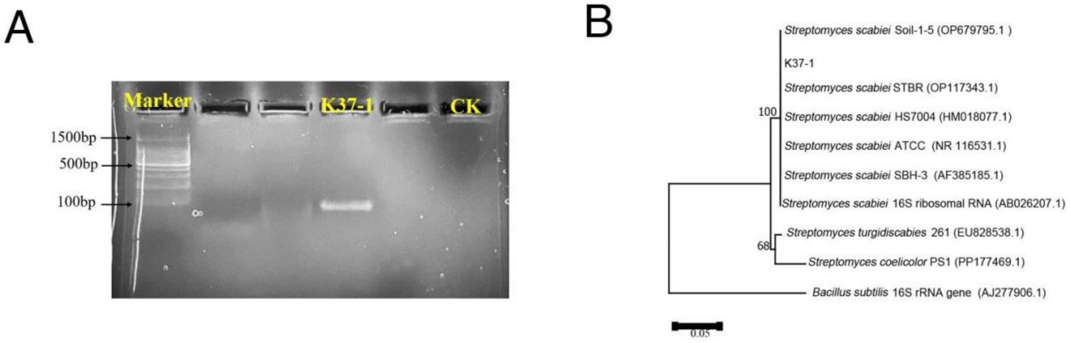

Supplementary Figure S1. Identification of the pathogenic strain *S. scabies* K37-1. (A) PCR amplification of the pathogenicity gene txtAB; (B) 16S rRNA gene-based phylogenetic tree.

1.2 Supplementary Table S1

Medium formulation

Name	Amount
Tryptic Soy Agar Medium	The medium consists of 17 g casein, 3 g peptone, 5 g sodium chloride, 2.5 g dipotassium hydrogen phosphate, 2.5 g glucose and 20 g agar, supplemented with distilled water to a final volume of 1 L
Tryptone-Yeast Extract-Glucose Agar	This solid medium is composed of 1 g tryptone, 1 g yeast extract, 0.5 g glucose, 6.34 g potassium chloride, 1.2 g sodium chloride, 0.25 g magnesium sulfate, 0.13 g dipotassium hydrogen phosphate, 0.22 g calcium chloride, 0.17 g potassium sulfate, 2.4 g sodium sulfate, 0.5 g sodium bicarbonate, 0.09 g sodium carbonate and 0.07 g Fe-EDTA. Add 20 g agar, and make up to 1 L with distilled water.
Yeast Extract Mannitol Agar	The medium is prepared with 0.5 g yeast extract, 5 g mannitol, 0.5 g dipotassium hydrogen phosphate, 0.2 g magnesium sulfate, 0.1 g sodium chloride and 20 g agar, and distilled water is added to bring the final volume to 1 L.
Rhizobium Agar Medium，M408	M408 solid medium consists of 1 g yeast extract, 10 g mannitol, 0.5 g dipotassium hydrogen phosphate, 0.2 g magnesium sulfate, 0.1 g sodium chloride and 20 g agar. Dissolve all ingredients and dilute with distilled water to a final volume of 1 L.

Yeast Mannitol Agar（M715）	M715 solid medium was prepared with 1 g yeast extract, 10 g mannitol, 0.5 g dipotassium hydrogen phosphate, 0.2 g magnesium sulfate, 0.1 g sodium chloride, 1 g calcium carbonate and 16 g agar, and distilled water was added to a final volume of 1 L.
Wheat Flour Medium	Flour solid medium contained 4 g pure wheat flour, 0.4 g calcium carbonate and 16 g agar, and distilled water was supplemented to a final volume of 1 L.
Tap Water-Yeast Extract Agar	Tap water yeast agar medium was prepared with 0.25 g yeast extract, 0.5 g dipotassium hydrogen phosphate and 18 g agar, and tap water was added to a final volume of 1 L.

1.3 Supplementary Figure S2

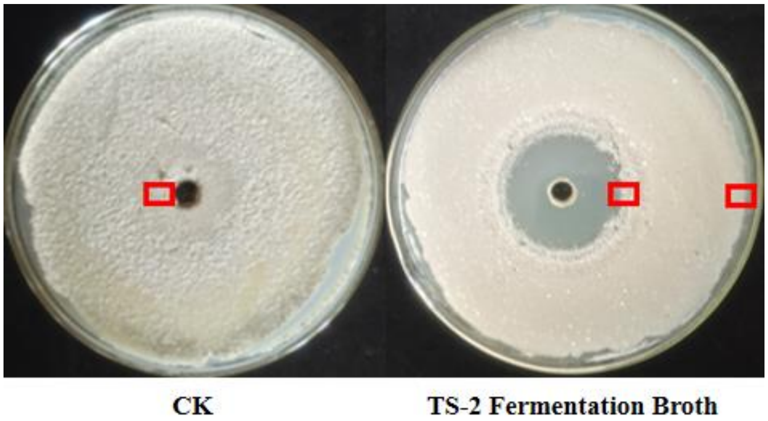

Supplementary Figure S2: The schematic diagram of scanning electron microscope and transcriptomics sampling site. The red part is the sampling area.

1.4 Supplementary Table S2

Mass Spectrometric Parameters

Description	Parameter
Scan type (m/z)	70-1050
Sheath gas flow rate(arb)	60
Aux gas flow rate(arb)	20
Heater temp (℃)	350

Capillary temp(°C)	320
Spray voltage(+)(V)	3400
Spray voltage(-) (V)	-3000
S-Lens RF Level	70
Normalized collision energy(%)	20,40,60
Resolution (Full MS)	60000
Resolution (MS^2)	15000

1.5 Supplementary Table S3

Genes and primers

Primer	Sequence（5' to 3'）
rpoB(F)	TCCCCGAGTCCTTCAAGGT
rpoB(R)	TGAGGCACAGGGACTGCAT
SCAB_RS03355(F)	GAGACCTTCCTCGACGTGTG
SCAB_RS03355(R)	CGTCAGTCCGTCGATCACC
SCAB_RS19060(F)	ACCACGACAGATACAGCACG
SCAB_RS19060(R)	CCGAAATCGAGGTCCACCC
SCAB_RS20980(F)	TCCAAGGTCCTCACCTCTCT
SCAB_RS20980(R)	CGATGTCAGCCGTGTAGGTG
SCAB_RS29870(F)	AACCGGACCGTCTACCACAT
SCAB_RS29870(R)	ATCGTGAGCAGCGAGTTGAC
SCAB_RS36015(F)	CGAATACGCCAAACAGCTCG
SCAB_RS36015(R)	GTCGTACCGGGAAGAAGCTC

SCAB_RS41120(F)	GAGTCGCTGGAGGATCTGC
SCAB_RS41120(R)	GCGGAAGTGCCTCTTGATCT
SCAB_RS17165(F)	TCTTCTTCCACCCCGAAGGA
SCAB_RS17165(R)	CTTGGCCGAGTTCTCACGA
SCAB_RS14030(F)	GGCTGTTCGTGATCATGCTG
SCAB_RS14030(R)	GAACGAGAAGAGGGACTGGG
SCAB_RS20620(F)	GCACCAGTCCCTGATCGTC
SCAB_RS20620(R)	GTCGTCGAACTGGAAGAGGC
SCAB_RS38735(F)	ATCTCGGGCACACCTGGTA
SCAB_RS38735(R)	GGCCCATACGGGAGAACAG
SCAB_RS20595(F)	TGGAGGTGGATCTCGACGG
SCAB_RS20595(R)	CGAGGTGGAAGACCATGCG
SCAB_RS37175(F)	GCACGAAACCCCATCTGAAC
SCAB_RS37175(R)	GCGATGTTGATGGTGATGCC
SCAB_RS05740(F)	GCACACAGCTACTTCACGTC
SCAB_RS05740(R)	TCGAGGCCGTTGAGAATGAA
SCAB_RS35735(F)	GAGTCCTCACTGCCCGTTC
SCAB_RS35735(R)	TTCGACCGTTTCCTCTGCTC

1.6 Supplementary Table S4

qRT-PCR System

Constituent	Volume

2×SYBR	5 μL
PCR Forward Primer(10 μM)	0.5 μL
PCR Reverse Primer(10 μM)	0.5 μL
cDNA	<100 ng
RNase-free H_2O	top up to10 μL

1.7 Supplementary Table S5

qRT-PCR amplification conditions

Order	Temperature	Time
Pre-denaturation	95℃	5 min
Denaturation	95℃	10 sec
Annealing	55℃	10 sec
Extension	72℃	20 sec
Final Extension	72℃	10 min

1.8 Supplementary Figure S3

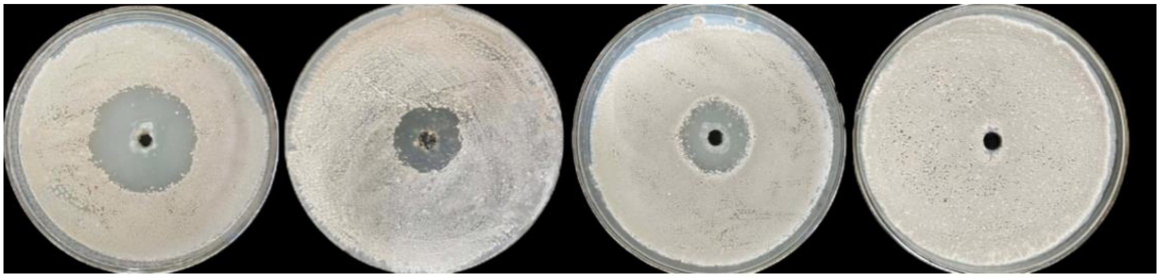

TS-2 TS-1 TS-3 CK

Supplementary Figure S3. Antagonistic activities of the antagonistic isolates TS-1, TS-2, and TS-3 against *S. scabies* in confrontation assays. CK: control without antagonistic bacterium.

1.9 Supplementary Figure S4

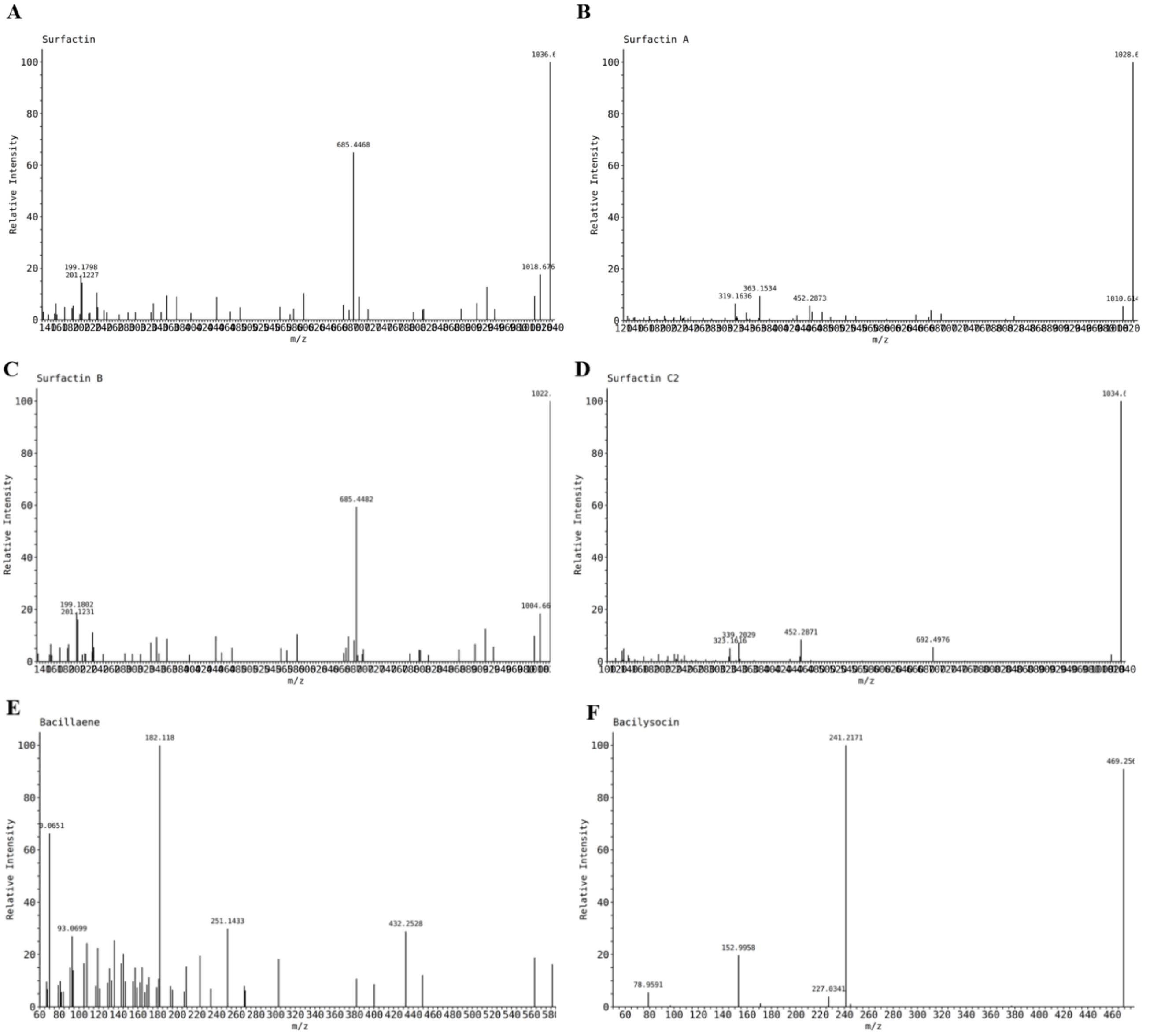

Supplementary Figure S4. Mass Spectra of Surfactin, Bacillaene, and Bacilysocin. (A-F) Represent the mass spectra of Surfactin, Surfactin A, Surfactin B, Surfactin C2, Bacillaene, and Bacilysocin, respectively.

1.10 Supplementary Table S6

GO Annotation of DEGs

GO term category	DEGS	Up	Dow n

regulation of biological process	57	17	40
small molecule metabolic process	141	36	105
biosynthetic process	154	68	86
nitrogen compound metabolic process	200	71	129
cellular metabolic process	227	76	151
primary metabolic process	231	70	161
organic substance metabolic process	300	91	209
membrane	285	106	179
catalytic activity, acting on a protein	54	22	32
transmembrane transporter activity	86	36	50
carbohydrate derivative binding	123	67	56
transferase activity	169	70	99
oxidoreductase activity	178	47	131
small molecule binding	206	109	97
hydrolase activity	231	83	148
ion binding	283	111	172
heterocyclic compound binding	309	140	169
organic cyclic compound binding	309	140	169

1.11 Supplementary Table S7

KEGG Annotation of DEGs

KEGG term category	DEGS	Up	Down
Transport and catabolism	9	-	8
Cell growth and death	11	3	8
Cellular community	19	5	14
Folding, sorting and degradation	7	4	3
Signal transduction	19	3	16
Membrane transport	47	25	22
Aging	7	3	4
Endocrine system	9	2	7
Glycan biosynthesis and metabolism	12	2	10
Global and overview maps	13	2	11
Metabolism of terpenoids and polyketides	13	3	10
Xenobiotics biodegradation and metabolism	19	3	16
Biosynthesis of other secondary metabolites	22	11	11
Lipid metabolism	23	-	22
Metabolism of other amino acids	26	4	22
Nucleotide metabolism	29	5	24
Metabolism of cofactors and vitamins	40	20	20
Energy metabolism	50	16	34
Carbohydrate metabolism	86	9	77
Amino acid metabolism	102	24	78
